# Music Therapy Interventions in Parkinson’s Disease: The State-of-the-Art

**DOI:** 10.3389/fneur.2015.00185

**Published:** 2015-08-31

**Authors:** Alfredo Raglio

**Affiliations:** ^1^Department of Public Health, Experimental and Forensic Medicine, University of Pavia, Pavia, Italy

**Keywords:** relational music therapy, neurological music therapy, rehabilitation, rhythm, motor symptoms, non-motor symptoms

Parkinson’s disease (PD) is a neurological disorder involving the progressive degeneration of the dopaminergic system, which gives rise to movement-related dysfunctions (such as bradykinesia, tremor, and rigidity) as well as other symptoms, mainly of cognitive and psychological nature. In the latter case, mood disorders prevails frequently causing anxiety and depression in all phases of the disease, sometimes even before the motor symptoms occur.

Aarsland and colleagues (1) report that 35% of the patients affected by PD present depression, whereas Richard (2) states that anxiety is to be found in 40% of the cases.

The literature shows that playing and listening to music may modulate emotions, behaviors, movements, communication, and cognitive factors, modifying the activity of the brain areas involved in the perception and regulation of these aspects (3, 4).

Music can produce substantial effects on movement-related symptoms as well as psychological ones in PD treatment. Concerning the first aspect, rhythm has a crucial role in rehabilitation, enhancing connections between the motor and auditory systems (5).

Literature showed how a rhythmic auditory cues-based training can produce a compensation of the cerebello-thalamo-cortical network leading to beneficial effects, for example, improving not only speed and step length but also perceptual and motor timing abilities (6, 7).

Areas involving rhythm perception are closely related to those that regulate movement (such as the premotor cortex, supplementary motor area, cerebellum, and basal ganglia – especially putamen) (8–18). A study conducted with fMRI (19) shows that whereas a regular pulse (in contrast to an irregular one) generally activates basal ganglia in a significant way, this is not the case in PD. Other studies (7, 20) support the idea that external cues (in particular rhythmical cues) can modulate the activity within the impaired timing system. This may mean that a regular rhythmic pulse stimulates the putamen activity, facilitating movement and providing an input for sequential movements and impaired automatized processes. Moreover, this could compensate for the lack of dopaminergic stimulation.

Rhythm can be also perceived visually and through the tactile sense, but the reaction time of the human auditory system is shorter by 20–50 ms, when compared to visual and tactile stimuli; moreover, it has a stronger capacity of perceiving rhythm periodicity and structure (6). Therefore, rhythm influences the kinetic system (through synchronization and adjustment of muscles to auditory stimuli), facilitates movement synchronization, coordination, and regularization, and may even produce an internal rhythm that persists in the absence of stimuli (21–23).

Many studies report that musical rhythm in PD treatment can improve gait (speed, frequency, and step length), limbs coordination, postural control, and balance (7, 18, 24–36). In view of the above, Neurologic Music Therapy (NMT) – especially Rhythmic Auditory Stimulation, one of its techniques – characterizes this approach to the disease: NMT aims at enhancing sensory, cognitive, and motor functions (as in PD treatment, in which specific rhythmic techniques can strengthen and improve the rehabilitative process).

Studies pertaining to non-motor symptoms (e.g., psychological aspects, such as anxiety or depression) are fewer in number, and therefore results are less certain (24, 36–41). From this point of view, music therapy approaches focused on the relationship between music therapist and patient serve as interventions that can produce substantial psychological improvements, creating moments of empathetic relationship and emotional connection. Sound is indeed an important means of communication, especially in a non-verbal context, promoting emotional expression and regulation. In PD treatment, the music therapeutic approach described above, based on free improvisation, is most assuredly less used, or maybe less documented. Sound/music improvisation can be considered as the main technique in the active music therapy approaches (42). During the improvisation, music therapist and patient interact freely using musical instruments, generally without musical rules or themes.

Moreover, making, and listening to, music can be considered as strong stimuli from the emotional point of view, playing an important role in the activation of the limbic system and neuro-chemical circuits (i.e., of the reward system) (43, 44).

A distinction between the relational and rehabilitative approach is extremely important, not only to determine the type of intervention but also to define the specific identity of the music therapy approach. In the former, the music therapist aims at building a relationship with the patient by means of interaction with melodic and rhythmical instruments and singing (improvisation). This facilitates the expression and modulation of patients’ emotions and promotes communication and empathetic relationships. In the latter, the music therapist proposes music-based exercises (in particular using rhythmical patterns) to improve motor, cognitive, and sensory functions, generally impaired by neurological damage. Although the two methods are often mutually influenced, there is a substantial difference in terms of goals, intervention models, techniques, and music therapy training. From this perspective, I believe that research should take these aspects into account, in order to focus on goals more accurately, while maintaining the possibility of overlapping the two approaches – making them complementary rather than antithetical. Often literature does not consider the distinction among these different music therapy approaches, defining every experience as “music therapy.” In contrast to this, I believe that the achievement of an adequate definition regarding different types of intervention is necessary; it should also highlight different theoretical and methodological bases and their different areas of application. To this end, a recent article by Raglio and Oasi (45) reports the main characteristics of music-based interventions, deriving from an in-depth analysis of their related contents and of literature.

Table 1 shows how clinical studies characterized by strong scientific implications (Randomized Controlled Trials and Clinical Controlled Trials) and based on rehabilitative treatments (not on evaluating tasks in the presence of a musical stimulus) are lacking in literature. The table summarizes the main effects produced on motor and non-motor symptoms and distinguishes the various interventions in terms of different musical contents and techniques.

**Table 1:** **Randomized Controlled Trials and Controlled Clinical Trials from PubMed database regarding Parkinson’s disease, including clinical studies (based on rehabilitative treatments) in English**.

Reference	Motor outcomes	Non-motor outcomes	Subjects *n* experimental group ***+*** *n* control group	Interventions/duration	Follow-up
(7)	Improving in perceptual and motor timing	Not evaluated	15 + 20	Rhythmic auditory cueing (beat + superimposed familiar songs)/4 weeks (30 min/session, 3 sessions/week)	Yes
(24)	Improving over time in motor function, cognitive function (verbal memory, language, and executive function and attention)	Slight improvement over time quality of life	12 + 6	Ronnie Gardiner rhythm and music (music method uses music, rhythm, movements and speech)/6 weeks (1 h/session, 2 sessions/week)	None
(25)	Improving in functional gait, balance, and freezing	Not evaluated	8 + 8	Rhythmic auditory stimulation (RAS)/6 weeks (45–60 min/session, 3 sessions/week)	Yes
(40)	Slight improvement over time in tremor	Improving over time in mood and anxiety; modest improvement on quality of life	18 + 18	Music relaxation/4 weeks (45 min/session, 2 sessions/week)	Yes
(41)	Significant improvement in bradykinesia	Improving over time in emotional functions, activities of daily living and quality of life	16 + 16	Music therapy sessions (choral singing, voice exercise, rhythmic and free body movements, and improvisational music therapy techniques)/3 months (2 h/session, weekly sessions)	Yes
(34)	Improving in gait parameters (velocity, stride length and step cadence)	Not evaluated	15 + 11	Rhythmic auditory stimulation (RAS) (beat + superimposed music)/3 weeks (30 min/session, daily)	None

*Search criteria: (“music” OR “music therapy” OR “rhythmic auditory stimulation”) AND “Parkinson*.”

In PD treatment based on NMT, it is important to understand what specifically acts on the disease: it is unclear, for example, whether the beat produces an effect by itself in neuromotor rehabilitation or, in contrast, the effects are produced by music (that we can consider the sum and combination of rhythm, melody, harmony, and other parameters) with a marked pulse (6). In fact, if music is excluded from interventions as described in many studies, we have to ask ourselves what then is the role of music therapists; furthermore, should such rhythm-based interventions be considered as part of music therapy programs, or rather simply as rehabilitative methods supported by rhythm, given that music does not assume a crucial role.

Additional weak points of NMT studies are given by the small sample size, the short duration of treatments and by the frequent lack of medium and long-term assessment (follow-up).

Another consideration is connected with the possibility to prove whether an effect produced during rehabilitation can persist also in the absence of stimuli. In this sense, the aforementioned studies conducted by Benjamin (21), Jackendoff (22), and Palmer and Krumhansl (23) offer various starting points that should be discussed in-depth, concerning the potential of the retrieval of imagined rhythm/music components, even after the stimuli have ceased or are absent (46).

A limited number of studies take the psychological outcome into account; these studies propose techniques based on a relational approach, which aims to develop an empathetic communication with the patient through the medium of sound. In music therapy, the active relational approach is based mainly on improvisation (42), and, from a practical standpoint, emphasizes the chances of enhancing and synchronizing the patient’s movements. When reviewing videotapes of the therapeutic sessions based on this approach, it becomes clear that there are significant changes in regularity and fluency of movement of the upper limbs that also positively influence the patient’s sound/music production. The music therapy relational approach in PD treatment is, therefore, less usual but equally as important; it is, in fact, less performance-centered and takes the patient’s personal expression into account, thus allowing a dynamic calibration of the stimulus, through a process of continuous regulation, combining and integrating the physical and psychological components. Considerations emerging from the relational music therapeutic experience in PD treatment emphasize the rhythmic component of improvisation, leading the patient into synchronization, at the same time maintaining freedom of expression and a non-prescriptive approach based on the free sonorous-music improvisation. This facilitates the integration of psychological aspects (the empathetic relationship, the regulation, and modulation of emotional expressions) with motor functions (speed and fluency of movement, in particular of the upper limbs).

Therefore, interventions that involve music can offer important starting points in PD rehabilitation, effectively acting on motor, as well as non-motor symptoms. In this sense, research should increase the number of studies based on strong methodological criteria, also including a clear description of the intervention – whether it be relational or rehabilitating – a consistent and numerically significant sample, in addition to more sensitive tools to evaluate motor and psychological outcomes. In conclusion, a stronger research methodology and a clearer definition of the exact medium or parameter in music related to specific output of rehabilitation are needed. This will allow the development of adequate, and increasingly specific and effective music therapy approaches.

## Conflict of Interest Statement

The author declares that the research was conducted in the absence of any commercial or financial relationships that could be construed as a potential conflict of interest.
